# Role of caspase-3 in development of neuronal plasticity and memory

**DOI:** 10.1186/2193-1801-4-S1-P57

**Published:** 2015-06-12

**Authors:** Igor Zhuravin, Nadezhda Dubrovskaya, Dmitrii Vasilev, Darya Kozlova, Natalia Tumanova, Anthony Turner, Natalia Nalivaeva

**Affiliations:** I.M. Institute of Evolutionary Physiology and Biochemistry, RAS, Russian Federation, Russia; Saint-Petersburg State Pediatric Medical University, Russian Federation, Russia; School of Molecular and Cellular Biology, University of Leeds, United Kingdom

**Keywords:** caspase-3, brain development, cognitive functions.

Caspases are known to play an important role in apoptosis and their increased activity in pre- and postsynaptic terminals leads to proteolysis of synapse-associated proteins, resulting in disruption of synaptic functions. Moreover, caspases degrade the C-terminal fragment of amyloid-precursor protein intracellular domain (AICD) which regulates expression of a variety of genes including an amyloid-degrading enzyme neprilysin (NEP). As such, inhibition of caspases is considered as a tool for prevention and compensation of various synaptic pathologies leading to cognitive deficit and Alzheimer’s disease pathogenesis. In this study we have evaluated the role of prenatal hypoxia on the activity of caspases and neuronal network characteristics in rat brain and the effect of caspase inhibitors on these parameters. We have found that the brain of rats subjected to prenatal hypoxia (Е14, О2 7%, 3 h) is characterised by an increased number of caspase-3-positive neurones and higher activity of this enzyme in the neocortex and hippocampus in the period of intensive synaptogenesis (Р20-30) compared to controls. Subsequently, in later life these animals had a reduced number of synaptopodin-positive dendritic spines and reduced activity of NEP accompanied by disruption of cognitive functions. Single i.v. injection of caspase-3 inhibitors (Ac-DEVD-CHO) to hypoxic rats on Р18-23 led to a decrease in caspase-3 activity and increased NEP expression. In these animals we have also observed restoration of synaptopodin levels and distribution of the labile synaptic spines in the neocortex and hippocampus which were accompanied by improved memory. The effects of inhibitors on memory was observed within one month after administration but not detected 2.5 months later. These data testify to the involvement of caspase-3 in normal brain development and indicate an important role of this enzyme in neuronal plasticity and regulation of cognitive functions.

Supported by RFBR (13-04-00388), ARUK.

